# Decyl­ammonium octa­noate

**DOI:** 10.1107/S1600536811005125

**Published:** 2011-02-19

**Authors:** Andrew E. Jefferson, Chenguang Sun, Andrew D. Bond, Stuart M. Clarke

**Affiliations:** a Department of Chemistry and BP Institute, University of Cambridge, Lensfield Road, Cambridge CB2 1EW, England; b Department of Physics and Chemistry, University of Southern Denmark, Campusvej 55, 5230 Odense, Denmark

## Abstract

The title compound, C_10_H_24_N^+^·C_8_H_15_O_2_^−^, forms a layered structure in which inter­molecular N^+^—H⋯O hydrogen bonds connect anions and cations, forming a two-dimensional network parallel to (010). The *n*-alkyl chains of the decyl­ammonium cations pack according to an ortho­rhom­bic ‘subcell’ with approximate dimensions 5.1 × 7.3 Å, and they are significantly distorted from planarity.

## Related literature

For background literature concerning compounds of alkyl carb­oxy­lic acids and primary alkyl amines, see: Backlund *et al.* (1994 ▶, 1997 ▶); Karlsson *et al.* (2000 ▶, 2001 ▶); Kohler *et al.* (1972 ▶); Kohler, Atrops, *et al.* (1981 ▶); Kohler, Gopal, *et al.* (1981 ▶). For a description of the ‘subcell’ associated with the packing of the *n*-alkyl chains, see: Dorset (2005 ▶).
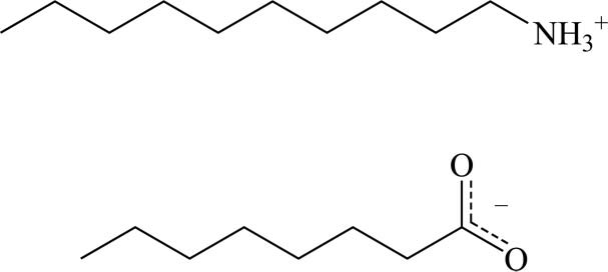


## Experimental

### 

#### Crystal data

C_10_H_24_N^+^·C_8_H_15_O_2_^−^
*M* *_r_* = 301.50
Monoclinic, 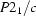

*a* = 5.5526 (2) Å
*b* = 44.489 (2) Å
*c* = 8.0931 (4) Å
β = 100.788 (3)°
*V* = 1963.90 (15) Å^3^
*Z* = 4
Mo *K*α radiation
μ = 0.06 mm^−1^
*T* = 180 K
0.35 × 0.18 × 0.02 mm

#### Data collection

Nonius KappaCCD diffractometer
Absorption correction: multi-scan (*SORTAV*; Blessing, 1995 ▶) *T*_min_ = 0.773, *T*_max_ = 1.000
5524 measured reflections
2233 independent reflections
1438 reflections with *I* > 2σ(*I*)
*R*_int_ = 0.053
θ_max_ = 22.0°

#### Refinement

*R*[*F* ^2^ > 2σ(*F* ^2^)] = 0.051
*wR*(*F* ^2^) = 0.128
*S* = 1.02
2233 reflections
202 parameters
3 restraints
H atoms treated by a mixture of independent and constrained refinement
Δρ_max_ = 0.13 e Å^−3^
Δρ_min_ = −0.17 e Å^−3^

### 

Data collection: *COLLECT* (Nonius, 1998 ▶); cell refinement: *SCALEPACK* (Otwinowski & Minor, 1997 ▶); data reduction: *DENZO* (Otwinowski & Minor, 1997 ▶) and *SCALEPACK*; program(s) used to solve structure: *SIR92* (Altomare *et al.*, 1994 ▶); program(s) used to refine structure: *SHELXL97* (Sheldrick, 2008 ▶); molecular graphics: *Mercury* (Macrae *et al.*, 2008 ▶); software used to prepare material for publication: *SHELXL97*.

## Supplementary Material

Crystal structure: contains datablocks global, I. DOI: 10.1107/S1600536811005125/lh5207sup1.cif

Structure factors: contains datablocks I. DOI: 10.1107/S1600536811005125/lh5207Isup2.hkl

Additional supplementary materials:  crystallographic information; 3D view; checkCIF report

## Figures and Tables

**Table 1:** Hydrogen-bond geometry (Å, °)

*D*—H⋯*A*	*D*—H	H⋯*A*	*D*⋯*A*	*D*—H⋯*A*
N1—H1*B*⋯O1	0.93 (1)	1.89 (1)	2.788 (3)	164 (2)
N1—H1*C*⋯O1^i^	0.92 (1)	1.91 (1)	2.821 (3)	170 (2)
N1—H1*A*⋯O2^ii^	0.92 (1)	1.85 (1)	2.768 (3)	175 (3)

Symmetry codes: (i) 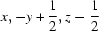; (ii) 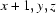.

**Fig. 1.**
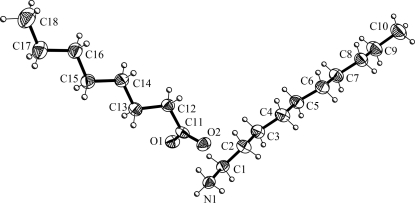
Molecular structure with displacement ellipsoids drawn at 50% probability for non-H atoms.

**Fig. 2.**
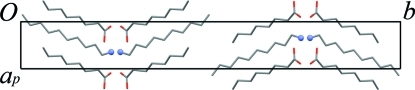
Projection along the c axis showing the layered structure. H atoms are omitted and the N atoms of the NH3+ groups are highlighted as spheres.

**Fig. 3.**
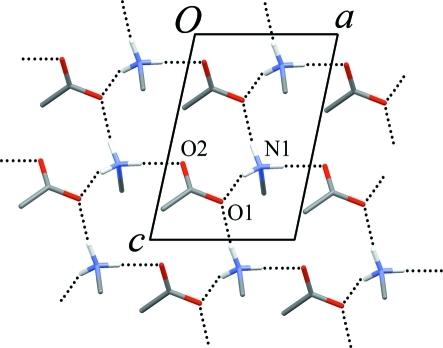
Section of the structure projected along the c axis, showing the hydrogen-bond topology (dashed lines). Only the C—CO2- and C—NH3+ groups are shown. All other C and H atoms are omitted.

**Fig. 4.**
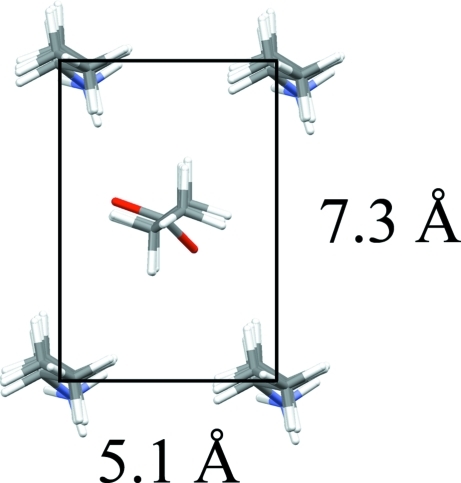
Section of the structure projected approximately along the long axes of the n-alkyl chains, showing the orthorhombic "subcell" packing. The dimensions indicated for the subcell are approximate. The third dimension of the subcell refers to the translational repeat of ca 2.54 Å along the n-alkyl chain. See Dorset (2005).

## supplementary crystallographic information

### Comment

The combination of alkyl carboxylic acids and primary alkyl amines is of continuing interest both in the bulk and in adsorbed monolayers. There is mainly spectroscopic evidence that a number of stoichiometric complexes can form, depending upon the molecular structure: combinations AB (1 acid: 1 amine), A_2_B and A_3_B have been reported (Backlund *et al.*, 1994; Backlund *et al.*, 1997; Karlsson *et al.*, 2000; Karlsson *et al.*, 2001; Kohler, Atrops *et al.*, 1981; Kohler, Gopal *et al.*, 1981; Kohler *et al.*, 1972). Interestingly, similar complexes have not been reported on the amine-rich side of the phase diagram. The precise nature of the complexation is still a matter of debate, but hydrogen bonding between the species is obviously strongly implicated and different structures have been proposed on this basis. However, we are not aware of any single-crystal diffraction studies for these materials.

The absence of reported single-crystal data for this class of complexes is probably attributable to difficulties in obtaining suitable crystals. Our various crystallization attempts have consistently failed, and our discovery of the crystal used for this study was serendipitous. The crystal was a thin plate that diffracted weakly, and data could be measured only to 0.95 Å resolution. Nonetheless, the data are adequate to localize the H atoms associated with the ammonium group, and these H atoms could be refined satisfactorily with restrained N—H bond lengths and individual isotropic displacement parameters. The C—O bond lengths of 1.269 (3) and 1.253 (3) Å are also consistent with proton transfer to yield a carboxylate anion. Both molecules adopt essentially fully extended conformations (*i.e.* the torsion angles along the main chain are all close to 180°), although the decylammonium chain is clearly disorted from planarity (Fig. 1). As a measure of this distortion, we note that the terminal C atom of the chain (C10) lies 1.43 (1) Å from the mean plane defined by atoms C1, C2 and C3.

As might be expected, the crystal structure is layered, with the hydrophilic sections accommodated around the glide planes parallel to (010) at *y* = 1/4 and 3/4 (Fig. 2). The hydrogen bonding between the ammonium groups and carboxylate anions (Table 1) defines a 2-D network comprising 6-membered rings (Fig. 3). Projection along the *n*-alkyl chains of the molecules reveals an approximately orthorhombic "subcell" with approximate dimensions 5.1 × 7.3 Å (the third dimension being the translation of *ca* 2.54 Å along the *n*-alkyl chain). The plane through the C atoms of the *n*-alkyl chain of each octanoate anion lies almost perpendicular to the planes of the *n*-alkyl chains of the ammonium cations (Fig. 4). This is a common subcell arrangement for long-chain *n*-alkyl compounds (Dorset, 2005). The distortion from planarity of the *n*-alkyl chain in the decylammonium cation serves to accommodate it between two neighbouring octanoic acid molecules [symmetry codes: 1 + *x*,0.5 - *y*,-1/2 + *z* and 1 + *x*,0.5 - *y*,1/2 + *z*], optimizing dispersion interactions along the length of the *n*-alkyl chains within the constraints imposed by the hydrogen-bonding geometry. At the interface between layers (*i.e.* in the (020) planes of the structure) the methyl groups of the decylammonium cations meet the methyl groups of the octanoate anions to form C···C contacts of 3.972 (4) Å, with the H atoms approximately eclipsed.

### Experimental

Octanoic acid (99%) and decylamine (99.5%) were obtained from Sigma Aldrich and used without further purification. A number of solution and melt methods were attempted to grow a single-crystal of sufficient dimensions and quality, but all were unsuccessful. A crystal was finally obtained serendipidously by growth from the vapour when poorly sealed vessels containing each of the individual components were stored together inside a small container (1 litre volume) in a glove bag initially purged with N_2_ and left undisturbed for a number of weeks. Crystal growth was observed on most of the plastic surfaces inside the storage container but principally on the polypropylene cap of the decylamine bottle. Elemental analysis found for the bulk sample: C 72.4, H 13.1, N, 4.8%; calculated C 71.7, H 13.0, N 4.7%.

### Refinement

The crystal diffracted relatively weakly, and data were collected to a maximum θ of 22° (0.95 Å resolution). Approximately 65% of data were observed at the 2σ level to this limit. The data are adequate to support location and refinement of the H atoms associated with the ammonium group. These were refined with N—H distances restrained to 0.91 (1) Å, and with individual *U*_iso_ values refined in the range 0.061 (10)–0.064 (10) Å^2^. All other H atoms were placed geometrically and refined as riding with C—H = 0.99 (CH_2_) or 0.98 (CH_3_) Å, and with *U*_iso_(H) = 1.2 or 1.5*U*_eq_(C).

### Crystal data

**Table:**

C_10_H_24_N^+^·C_8_H_15_O_2_^−^	*F*(000) = 680
*M**_r_* = 301.50	*D*_x_ = 1.020 Mg m^−^^3^
Monoclinic, *P*2_1_/*c*	Mo *K*α radiation, λ = 0.71073 Å
Hall symbol: -P 2ybc	Cell parameters from 29938 reflections
*a* = 5.5526 (2) Å	θ = 1.0–22.0°
*b* = 44.489 (2) Å	µ = 0.06 mm^−^^1^
*c* = 8.0931 (4) Å	*T* = 180 K
β = 100.788 (3)°	Block, colourless
*V* = 1963.90 (15) Å^3^	0.35 × 0.18 × 0.02 mm
*Z* = 4	

### Data collection

**Table:**

Nonius KappaCCD diffractometer	1438 reflections with *I* > 2σ(*I*)
Radiation source: fine-focus sealed tube	*R*_int_ = 0.053
ω and φ scans	θ_max_ = 22.0°, θ_min_ = 3.7°
Absorption correction: multi-scan (*SORTAV*; Blessing, 1995)	*h* = −5→5
*T*_min_ = 0.773, *T*_max_ = 1.000	*k* = −46→46
5524 measured reflections	*l* = −8→8
2233 independent reflections	

### Refinement

**Table:**

Refinement on *F*^2^	Primary atom site location: structure-invariant direct methods
Least-squares matrix: full	Secondary atom site location: difference Fourier map
*R*[*F*^2^ > 2σ(*F*^2^)] = 0.051	Hydrogen site location: inferred from neighbouring sites
*wR*(*F*^2^) = 0.128	H atoms treated by a mixture of independent and constrained refinement
*S* = 1.02	*w* = 1/[σ^2^(*F*_o_^2^) + (0.0647*P*)^2^] where *P* = (*F*_o_^2^ + 2*F*_c_^2^)/3
2233 reflections	(Δ/σ)_max_ < 0.001
202 parameters	Δρ_max_ = 0.13 e Å^−^^3^
3 restraints	Δρ_min_ = −0.17 e Å^−^^3^

### Special details

**Table:**

Geometry. All e.s.d.'s (except the e.s.d. in the dihedral angle between two l.s. planes) are estimated using the full covariance matrix. The cell e.s.d.'s are taken into account individually in the estimation of e.s.d.'s in distances, angles and torsion angles; correlations between e.s.d.'s in cell parameters are only used when they are defined by crystal symmetry. An approximate (isotropic) treatment of cell e.s.d.'s is used for estimating e.s.d.'s involving l.s. planes.
Refinement. Refinement of *F*^2^ against ALL reflections. The weighted *R*-factor *wR* and goodness of fit *S* are based on *F*^2^, conventional *R*-factors *R* are based on *F*, with *F* set to zero for negative *F*^2^. The threshold expression of *F*^2^ > σ(*F*^2^) is used only for calculating *R*-factors(gt) *etc*. and is not relevant to the choice of reflections for refinement. *R*-factors based on *F*^2^ are statistically about twice as large as those based on *F*, and *R*- factors based on ALL data will be even larger.

### Fractional atomic coordinates and isotropic or equivalent isotropic displacement parameters (Å^2^)

**Table:**

	*x*	*y*	*z*	*U*_iso_*/*U*_eq_	
N1	0.6509 (4)	0.23800 (6)	0.6408 (3)	0.0368 (6)	
H1A	0.797 (3)	0.2475 (6)	0.635 (3)	0.064 (10)*	
H1B	0.552 (4)	0.2520 (5)	0.681 (3)	0.061 (10)*	
H1C	0.564 (4)	0.2308 (6)	0.540 (2)	0.063 (10)*	
C1	0.7131 (5)	0.21366 (6)	0.7683 (3)	0.0376 (7)	
H1D	0.7889	0.2226	0.8776	0.045*	
H1E	0.8346	0.2000	0.7330	0.045*	
C2	0.4907 (5)	0.19598 (6)	0.7896 (3)	0.0443 (8)	
H2A	0.4128	0.1875	0.6794	0.053*	
H2B	0.3712	0.2097	0.8271	0.053*	
C3	0.5494 (5)	0.17059 (6)	0.9156 (3)	0.0453 (8)	
H3A	0.6910	0.1591	0.8902	0.054*	
H3B	0.5984	0.1793	1.0296	0.054*	
C4	0.3373 (5)	0.14915 (6)	0.9157 (3)	0.0479 (8)	
H4A	0.1991	0.1606	0.9465	0.057*	
H4B	0.2827	0.1415	0.7999	0.057*	
C5	0.3925 (5)	0.12257 (7)	1.0335 (3)	0.0469 (8)	
H5A	0.4359	0.1301	1.1504	0.056*	
H5B	0.5369	0.1118	1.0077	0.056*	
C6	0.1810 (5)	0.10059 (6)	1.0222 (3)	0.0466 (8)	
H6A	0.0387	0.1113	1.0520	0.056*	
H6B	0.1335	0.0938	0.9042	0.056*	
C7	0.2351 (5)	0.07329 (7)	1.1342 (4)	0.0511 (8)	
H7A	0.2901	0.0801	1.2516	0.061*	
H7B	0.3725	0.0621	1.1009	0.061*	
C8	0.0211 (5)	0.05205 (6)	1.1290 (4)	0.0505 (8)	
H8A	−0.1158	0.0633	1.1630	0.061*	
H8B	−0.0345	0.0453	1.0114	0.061*	
C9	0.0743 (6)	0.02465 (7)	1.2396 (4)	0.0648 (10)	
H9A	0.1386	0.0313	1.3563	0.078*	
H9B	0.2044	0.0128	1.2015	0.078*	
C10	−0.1459 (6)	0.00445 (7)	1.2403 (4)	0.0763 (11)	
H10A	−0.0969	−0.0128	1.3145	0.114*	
H10B	−0.2086	−0.0027	1.1258	0.114*	
H10C	−0.2744	0.0158	1.2809	0.114*	
O1	0.4243 (3)	0.28030 (4)	0.81491 (19)	0.0384 (5)	
O2	0.1023 (3)	0.26380 (4)	0.6328 (2)	0.0424 (5)	
C11	0.1952 (5)	0.27876 (6)	0.7601 (3)	0.0333 (7)	
C12	0.0316 (4)	0.29610 (6)	0.8566 (3)	0.0358 (7)	
H12A	0.0647	0.2891	0.9748	0.043*	
H12B	−0.1413	0.2912	0.8086	0.043*	
C13	0.0628 (5)	0.33012 (6)	0.8553 (3)	0.0368 (7)	
H13A	0.2345	0.3353	0.9050	0.044*	
H13B	0.0292	0.3374	0.7376	0.044*	
C14	−0.1081 (5)	0.34575 (6)	0.9533 (3)	0.0400 (7)	
H14A	−0.0733	0.3381	1.0702	0.048*	
H14B	−0.2785	0.3400	0.9038	0.048*	
C15	−0.0940 (5)	0.37981 (6)	0.9594 (3)	0.0419 (8)	
H15A	0.0749	0.3859	1.0110	0.050*	
H15B	−0.1288	0.3877	0.8431	0.050*	
C16	−0.2713 (5)	0.39370 (6)	1.0579 (3)	0.0447 (8)	
H16A	−0.2394	0.3851	1.1728	0.054*	
H16B	−0.4400	0.3879	1.0041	0.054*	
C17	−0.2585 (5)	0.42763 (6)	1.0715 (4)	0.0527 (8)	
H17A	−0.2878	0.4363	0.9568	0.063*	
H17B	−0.0911	0.4335	1.1277	0.063*	
C18	−0.4416 (5)	0.44107 (7)	1.1682 (4)	0.0704 (10)	
H18A	−0.4233	0.4630	1.1721	0.106*	
H18B	−0.4115	0.4330	1.2830	0.106*	
H18C	−0.6084	0.4359	1.1119	0.106*	

### Atomic displacement parameters (Å^2^)

**Table:**

	*U*^11^	*U*^22^	*U*^33^	*U*^12^	*U*^13^	*U*^23^
N1	0.0325 (15)	0.0405 (16)	0.0377 (16)	−0.0004 (14)	0.0076 (13)	−0.0051 (14)
C1	0.0383 (16)	0.0406 (18)	0.0332 (15)	0.0040 (15)	0.0051 (12)	0.0004 (15)
C2	0.0379 (17)	0.051 (2)	0.0441 (17)	−0.0020 (16)	0.0070 (13)	0.0085 (16)
C3	0.0410 (17)	0.052 (2)	0.0412 (17)	−0.0032 (16)	0.0038 (14)	0.0024 (16)
C4	0.0411 (17)	0.059 (2)	0.0427 (18)	−0.0047 (17)	0.0053 (14)	0.0068 (17)
C5	0.0435 (18)	0.052 (2)	0.0449 (17)	−0.0015 (16)	0.0069 (14)	0.0087 (17)
C6	0.0470 (18)	0.048 (2)	0.0448 (18)	0.0012 (16)	0.0081 (14)	0.0064 (16)
C7	0.0499 (19)	0.049 (2)	0.0553 (19)	0.0005 (16)	0.0115 (15)	0.0081 (17)
C8	0.0524 (19)	0.045 (2)	0.056 (2)	−0.0044 (17)	0.0152 (15)	0.0033 (17)
C9	0.067 (2)	0.056 (2)	0.076 (2)	−0.001 (2)	0.0242 (18)	0.013 (2)
C10	0.080 (3)	0.057 (2)	0.100 (3)	−0.010 (2)	0.036 (2)	0.007 (2)
O1	0.0247 (11)	0.0510 (13)	0.0390 (10)	0.0009 (9)	0.0048 (8)	−0.0016 (10)
O2	0.0360 (11)	0.0542 (14)	0.0364 (11)	−0.0062 (10)	0.0049 (9)	−0.0129 (11)
C11	0.0300 (17)	0.0360 (18)	0.0349 (16)	0.0013 (15)	0.0084 (13)	0.0116 (16)
C12	0.0306 (15)	0.0392 (18)	0.0381 (16)	−0.0013 (14)	0.0080 (13)	−0.0008 (14)
C13	0.0333 (15)	0.0378 (18)	0.0392 (16)	−0.0006 (14)	0.0065 (12)	0.0036 (14)
C14	0.0389 (16)	0.039 (2)	0.0441 (16)	0.0028 (15)	0.0124 (13)	0.0000 (15)
C15	0.0389 (17)	0.041 (2)	0.0472 (17)	0.0004 (15)	0.0105 (14)	−0.0029 (15)
C16	0.0452 (18)	0.040 (2)	0.0492 (18)	0.0021 (16)	0.0095 (14)	−0.0030 (16)
C17	0.0494 (19)	0.045 (2)	0.063 (2)	0.0036 (17)	0.0077 (16)	−0.0066 (17)
C18	0.065 (2)	0.059 (2)	0.088 (3)	0.0103 (19)	0.0179 (19)	−0.015 (2)

### Geometric parameters (Å, °)

**Table:**

N1—C1	1.491 (3)	C9—H9A	0.990
N1—H1A	0.92 (1)	C9—H9B	0.990
N1—H1B	0.93 (1)	C10—H10A	0.980
N1—H1C	0.92 (1)	C10—H10B	0.980
C1—C2	1.501 (3)	C10—H10C	0.980
C1—H1D	0.990	O1—C11	1.268 (3)
C1—H1E	0.990	O2—C11	1.253 (3)
C2—C3	1.516 (3)	C11—C12	1.515 (3)
C2—H2A	0.990	C12—C13	1.524 (3)
C2—H2B	0.990	C12—H12A	0.990
C3—C4	1.516 (3)	C12—H12B	0.990
C3—H3A	0.990	C13—C14	1.515 (3)
C3—H3B	0.990	C13—H13A	0.990
C4—C5	1.514 (4)	C13—H13B	0.990
C4—H4A	0.990	C14—C15	1.517 (3)
C4—H4B	0.990	C14—H14A	0.990
C5—C6	1.518 (3)	C14—H14B	0.990
C5—H5A	0.990	C15—C16	1.510 (3)
C5—H5B	0.990	C15—H15A	0.990
C6—C7	1.511 (4)	C15—H15B	0.990
C6—H6A	0.990	C16—C17	1.514 (4)
C6—H6B	0.990	C16—H16A	0.990
C7—C8	1.513 (4)	C16—H16B	0.990
C7—H7A	0.990	C17—C18	1.517 (4)
C7—H7B	0.990	C17—H17A	0.990
C8—C9	1.508 (4)	C17—H17B	0.990
C8—H8A	0.990	C18—H18A	0.980
C8—H8B	0.990	C18—H18B	0.980
C9—C10	1.518 (4)	C18—H18C	0.980
			
C1—N1—H1A	105.9 (17)	C8—C9—H9B	108.7
C1—N1—H1B	108.7 (18)	C10—C9—H9B	108.7
H1A—N1—H1B	107 (3)	H9A—C9—H9B	107.6
C1—N1—H1C	111.8 (18)	C9—C10—H10A	109.5
H1A—N1—H1C	116 (2)	C18^i^—C10—H10A	53.7
H1B—N1—H1C	107 (2)	C9—C10—H10B	109.5
N1—C1—C2	111.8 (2)	C18^i^—C10—H10B	79.7
N1—C1—H1D	109.3	H10A—C10—H10B	109.5
C2—C1—H1D	109.3	C9—C10—H10C	109.5
N1—C1—H1E	109.3	H10A—C10—H10C	109.5
C2—C1—H1E	109.3	H10B—C10—H10C	109.5
H1D—C1—H1E	107.9	O2—C11—O1	123.2 (2)
C1—C2—C3	112.9 (2)	O2—C11—C12	120.0 (2)
C1—C2—H2A	109.0	O1—C11—C12	116.8 (3)
C3—C2—H2A	109.0	C11—C12—C13	115.0 (2)
C1—C2—H2B	109.0	C11—C12—H12A	108.5
C3—C2—H2B	109.0	C13—C12—H12A	108.5
H2A—C2—H2B	107.8	C11—C12—H12B	108.5
C4—C3—C2	113.6 (2)	C13—C12—H12B	108.5
C4—C3—H3A	108.9	H12A—C12—H12B	107.5
C2—C3—H3A	108.9	C14—C13—C12	111.7 (2)
C4—C3—H3B	108.9	C14—C13—H13A	109.3
C2—C3—H3B	108.9	C12—C13—H13A	109.3
H3A—C3—H3B	107.7	C14—C13—H13B	109.3
C5—C4—C3	115.2 (2)	C12—C13—H13B	109.3
C5—C4—H4A	108.5	H13A—C13—H13B	107.9
C3—C4—H4A	108.5	C13—C14—C15	116.2 (2)
C5—C4—H4B	108.5	C13—C14—H14A	108.2
C3—C4—H4B	108.5	C15—C14—H14A	108.2
H4A—C4—H4B	107.5	C13—C14—H14B	108.2
C4—C5—C6	113.7 (2)	C15—C14—H14B	108.2
C4—C5—H5A	108.8	H14A—C14—H14B	107.4
C6—C5—H5A	108.8	C16—C15—C14	113.0 (2)
C4—C5—H5B	108.8	C16—C15—H15A	109.0
C6—C5—H5B	108.8	C14—C15—H15A	109.0
H5A—C5—H5B	107.7	C16—C15—H15B	109.0
C7—C6—C5	114.6 (2)	C14—C15—H15B	109.0
C7—C6—H6A	108.6	H15A—C15—H15B	107.8
C5—C6—H6A	108.6	C15—C16—C17	114.8 (2)
C7—C6—H6B	108.6	C15—C16—H16A	108.6
C5—C6—H6B	108.6	C17—C16—H16A	108.6
H6A—C6—H6B	107.6	C15—C16—H16B	108.6
C6—C7—C8	114.7 (2)	C17—C16—H16B	108.6
C6—C7—H7A	108.6	H16A—C16—H16B	107.5
C8—C7—H7A	108.6	C16—C17—C18	113.8 (2)
C6—C7—H7B	108.6	C16—C17—H17A	108.8
C8—C7—H7B	108.6	C18—C17—H17A	108.8
H7A—C7—H7B	107.6	C16—C17—H17B	108.8
C9—C8—C7	115.0 (2)	C18—C17—H17B	108.8
C9—C8—H8A	108.5	H17A—C17—H17B	107.7
C7—C8—H8A	108.5	C17—C18—H18A	109.5
C9—C8—H8B	108.5	C17—C18—H18B	109.5
C7—C8—H8B	108.5	H18A—C18—H18B	109.5
H8A—C8—H8B	107.5	C17—C18—H18C	109.5
C8—C9—C10	114.3 (3)	H18A—C18—H18C	109.5
C8—C9—H9A	108.7	H18B—C18—H18C	109.5
C10—C9—H9A	108.7		
			
N1—C1—C2—C3	178.6 (2)	O2—C11—C12—C13	−115.7 (3)
C1—C2—C3—C4	−169.5 (2)	O1—C11—C12—C13	64.4 (3)
C2—C3—C4—C5	177.0 (2)	C11—C12—C13—C14	179.6 (2)
C3—C4—C5—C6	−176.4 (2)	C12—C13—C14—C15	−179.6 (2)
C4—C5—C6—C7	177.9 (2)	C13—C14—C15—C16	179.4 (2)
C5—C6—C7—C8	177.4 (2)	C14—C15—C16—C17	178.3 (2)
C6—C7—C8—C9	179.6 (2)	C15—C16—C17—C18	178.9 (2)
C7—C8—C9—C10	176.8 (3)		

Symmetry codes: (i) −*x*−1, *y*−1/2, −*z*+5/2.

### Hydrogen-bond geometry (Å, °)

**Table:**

*D*—H···*A*	*D*—H	H···*A*	*D*···*A*	*D*—H···*A*
N1—H1B···O1	0.93 (1)	1.89 (1)	2.788 (3)	164 (2)
N1—H1C···O1^ii^	0.92 (1)	1.91 (1)	2.821 (3)	170 (2)
N1—H1A···O2^iii^	0.92 (1)	1.85 (1)	2.768 (3)	175 (3)

Symmetry codes: (ii) *x*, −*y*+1/2, *z*−1/2; (iii) *x*+1, *y*, *z*.
